# Quantifying Epidemiological Risk Transitions of COVID-19 in the Brazilian State of Ceará (2020–2023): A Generalized Linear Modeling Approach

**DOI:** 10.3390/epidemiologia7030083

**Published:** 2026-06-15

**Authors:** Matheus Paiva Emidio Cavalcanti, Carlos Mendes Tavares, Yasmin Esther Barreto, Alexandre Castelo Branco Araujo, Rosalina Semedo de Andrade, Luiz Carlos de Abreu

**Affiliations:** 1 COVID-19 Observatory Brazil and Ireland, School of Medicine, University of Limerick, V94 T9PX Limerick, Ireland; jhgd.marilia@unesp.br; 2 Postgraduate Program in Medical Sciences, Faculty of Medicine, University of São Paulo, Sao Paulo 01246-903, SP, Brazil; alexandrecbranco@terra.com.br; 3 Institute of Applied Social Sciences, University of International Integration of Afro-Brazilian Lusophony (UNILAB), Redenção 62790-000, CE, Brazil; carlostavares@unilab.edu.br (C.M.T.); rosalina@unilab.edu.br (R.S.d.A.); 4 Laboratory of Design of Studies and Scientific Writing, Federal University of Espirito Santo, Vitória 29075-910, ES, Brazil; 5 Research Scholar, Rehabilitation Sciences, Florida Gulf Coast University (FGCU), 10501 FGCU Blvd. S., Fort Myers, FL 33965, USA

**Keywords:** COVID-19, health transition, epidemiological monitoring, time series studies, Linear Models

## Abstract

Background/Objectives: While the descriptive trajectory of COVID-19 is well-documented, there is a methodological gap in quantifying the precise magnitude of risk reduction across multi-year pandemic phases in Brazilian subnational units. This study aimed to fill this gap by applying Generalized Linear Models (GLMs) to quantify the temporal transition of epidemiological risks (Incidence, Mortality, and Case Fatality) in Ceará (2020–2023), using the first year of the pandemic as a statistical baseline. Methods: Ecological time-series study was conducted using official surveillance data. We employed GLMs with Poisson distribution to calculate Rate Ratios (RRs) and 95% Confidence Intervals, allowing for a robust comparative risk modeling between 2020 (reference) and subsequent years (2021–2023). Results: Modeling revealed a significant epidemiological dissociation between transmission and severity. While the risk of incidence remained high through 2022 (RR = 1.42), the mortality risk showed an earlier and more drastic decline, with a 68% reduction as early as 2022 (RR = 0.32) and 99% in 2023 (RR = 0.01). The Case Fatality Rate (CFR) risk decreased consistently from 2021 onwards, reaching its lowest point in 2023 (RR = 0.09; 91% reduction). Conclusions: Between 2020 and 2023, Ceará transitioned to reduced COVID-19 severity. Despite ecological design and data limitations, these findings underscore the importance of resilient health systems and equitable immunization.

## 1. Introduction

The global emergence of Severe Acute Respiratory Syndrome Coronavirus 2 (SARS-CoV-2) in early 2020 triggered the most serious health crisis of the century, marked by unprecedented morbidity and mortality [1]. The evolution of the COVID-19 pandemic internationally was characterized by distinct phases: an initial period of high transmissibility and Case Fatality in 2020 [2], the emergence of variants of concern that amplified subsequent waves in 2021, and a gradual transition to endemicity from 2022 onwards [3]. In Brazil, a country of continental dimensions and marked socioeconomic heterogeneity, the clash with COVID-19 imposed complex challenges on public health systems [4,5,6,7].

The initial emergency response evolved into strategies focused on mitigating severe outcomes, with vaccination being the main pillar for containing the progression of the disease in scenarios of persistent viral circulation [8,9,10,11]. Despite the vast literature on the global trajectory of the pandemic, a gap remains in understanding the specific and quantified dynamics in large subnational units, such as the Brazilian states, which implemented health policies, experiencing diverse epidemiological outcomes, with varying degrees of success [12,13,14].

Most studies focus on descriptive analysis of peaks or short periods of the pandemic periods. What is still largely unknown is the precise and statistically robust magnitude of risk reduction throughout the entire time series (2020–2023), particularly regarding the epidemiological dissociation between mortality and incidence, a phenomenon where the risk of death diverges from the risk of infection [15,16].

The Brazilian state of Ceará, as a hub for epidemiological surveillance and hospital care in the Northeast region, offers a relevant scenario for evaluating these transitions [17]. Understanding how much and when this risk reduction became statistically significant in Ceará is essential for characterizing regional epidemiological behavior. To address this methodological gap, this study employs a robust comparative risk modeling approach using Generalized Linear Models (GLMs) [18] with Poisson distribution [19].

This methodological choice allows for modeling count data subject to overdispersion, an intrinsic characteristic of pandemic notification data, and quantifying, through the Rate Ratio (RR), the variation in epidemiological risk in each subsequent year compared to the 2020 as baseline year. Thus, the method transcends descriptive analysis, providing high-rigor scientific metric to compare the intensity of the pandemic across its different phases.

Therefore, the aim of this work is to analyze the long-term temporal trends of key epidemiological indicators of COVID-19 (Incidence Rate, Mortality Rate, and Case Fatality Ratio) in the Brazilian state of Ceará, from 2020 to 2023, and to quantify the magnitude of risk reduction in each phase of the pandemic through statistical time series modeling.

## 2. Materials and Methods

### 2.1. Study Design and Data Sources

This work is characterized as an ecological study based on aggregated secondary data [20,21], conducted with the objective to analyze the temporal evolution of the main epidemiological indicators of COVID-19 in the state of Ceará, Brazil, throughout the period from 2020 to 2023, covering from the beginning of SARS-CoV-2 circulation in the state of Ceará to the subsequent phase of greater epidemiological stabilization, observed after the wide implementation of vaccination strategies and the progressive reduction in population risk associated with COVID-19. The ecological design was adopted for its suitability in assessing population-level trends and comparing distinct epidemiological periods, while explicitly acknowledging its inherent limitations regarding individual-level causal inference.

Official secondary data were obtained from the public COVID-19 database in Brazil’s national epidemiological surveillance systems, provided by the Brazilian Ministry of Health consolidated database through the website: https://covid.saude.gov.br/ (accessed 10 February 2024) [22]. Time series analysis helps organize quantitative information distributed over time, allowing for the understanding, prediction, and remediation of potential future disease scenarios amidst a population. Population estimates used as denominators for rate calculations were extracted from the Brazilian Institute of Geography and Statistics (IBGE), considering annual projections specific to the state of Ceará [23].

### 2.2. Definition of Variables and Epidemiological Indicators

After aggregating the data, three standardized indicators were constructed, defined as the dependent variable of the study: the Incidence Rate (IR), calculated as the number of confirmed cases divided by the resident population per 100,000 inhabitants; the Mortality Rate (MR), determined by the ratio between the number of confirmed deaths and the resident population, also expressed per 100,000 inhabitants; and the Case Fatality Rate (CFR), representing the percentage proportion of deaths in relation to the total number of confirmed cases. The formulas used were:

Incidence = (New cases of COVID-19 in a given period)/(population in a given period × 100,000)

Mortality = (COVID-19 deaths in a given period)/(population in a given period × 100,000)

Case Fatality = (COVID-19 deaths in a given period)/(New cases of COVID-19 in a given period) × 100

The main independent variable was the calendar year, treated as a categorical factor to allow for a comparative analysis of the epidemiological risk in each period relative to the 2020 baseline.

### 2.3. Data Processing and Inclusion Criteria

All official records of confirmed cases and deaths attributed to COVID-19 occurring between 1 January 2020, and 31 December 2023, among residents of Ceará were included in the analysis. Records classified as duplicates, inconsistent, or lacking essential epidemiological information were managed according to the standardized validation and data-cleaning procedures implemented by the official health information systems from which the databases were obtained.

The present study relied exclusively on consolidated secondary data; no additional arbitrary exclusion criteria, case-level exclusions or modifications were performed by the authors beyond those procedures already applied by the surveillance systems. To assess the potential influence of these automated validation procedures on the robustness of the estimates, a sensitivity assessment was conducted comparing aggregated indicators before and after the exclusion of flagged records. This procedure confirmed that the data-cleaning process did not produce meaningful changes in the magnitude or direction of the final estimates.

The basic unit of observation consisted of the daily records of COVID-19 cases and deaths. For analytical purposes, data were aggregated at two temporal scales: monthly and annual. The monthly aggregation was used to characterize temporal variability and calculate descriptive statistics, whereas the annual aggregation was employed for the calculation of standardized epidemiological rates and for the comparative statistical modeling between years.

### 2.4. Statistical Modeling and Temporal Inference

The primary inferential analysis was based on fitting Generalized Linear Models (GLMs) [18] with a log link function and Poisson distribution, an approach appropriate for modeling count data and rates in population-level epidemiological studies [19]. The dependent variables corresponded to the annual number of confirmed cases and deaths due to COVID-19, while calendar year was included as a categorical independent variable, allowing direct estimation of rate ratios (Rate Ratios—RRs) associated with each period, using the year 2020 as the reference category.

The adequacy of the Poisson distribution was supported by the absence of statistically relevant overdispersion, assessed through the ratio between residual deviance and the degrees of freedom of the fitted models, as consolidated practice in count data modeling. When marginal deviations from this condition were observed, they were interpreted as inherent limitations of aggregated data analysis, without compromising the validity of relative estimates. This approach was chosen to model the annual counts of cases and deaths, quantifying the variation in risk through Rate Ratios (RRs), with 2020 defined as the reference year. By using 2020 as a baseline, the GLM-Poisson model respects the temporal structure of the data and provides a statistically valid estimation of risk transitions through Rate Ratios (RRs), addressing the inherent dependencies in the time series.

In all fitted models, this parameter remained close to unity (Φ ≈ 1.0), indicating the absence of relevant overdispersion. This result confirms the adequacy of the Poisson Generalized Linear Model for the analyzed data, with no empirical evidence justifying the adoption of alternative models with expanded variance structures, such as the Negative Binomial model.

As an additional verification procedure, we also evaluated the stability of the estimates under different model specifications, observing that the estimated rate ratios remained consistent, reinforcing the robustness of the adopted approach. All estimates were presented as Rate Ratios (RRs), accompanied by 95% confidence intervals (95% CI) [18,19,24].

### 2.5. Analytical Rationale and Proxy Variables

Within this ecological framework, the calendar year was utilized as a proxy variable. This choice captures the aggregated and temporally structured effects of multiple factors, including the expansion of vaccination, the accumulation of combined immunity, and changes in diagnostic practices. This approach is consistent with the goal of estimating relative risk variations at the population level without the intention of mechanistic decomposition of individual risk factors.

Regarding the modeling framework, the annual resident population was utilized as a fixed offset (denominator) to ensure stability and comparability of the rates across the study period. While adjusting the population-at-risk by subtracting individuals with immunity from prior infection or vaccination was conceptually considered, such an approach would require multiple unverifiable assumptions at the subnational level.

These include arbitrary parameters for immunity waning, heterogeneous vaccine effectiveness against different variants, and the lack of harmonized daily data on hybrid immunity prevalence in Ceará. Therefore, maintaining a stable denominator based on official census data was preferred to avoid introducing structural biases that could compromise the inferential robustness of the Rate Ratios (RRs).

It is acknowledged that COVID-19 dynamics are influenced by factors such as vaccination coverage, immunity acquired through prior infection, circulation of viral variants, and availability of diagnostic testing [25]. However, within an ecological study based on aggregated official data, such determinants are not observable at the individual level and act as structural components implicitly reflected in the temporal variations in the analyzed epidemiological indicators.

Additionally, retrospective harmonized and continuous estimates of the number of protected individuals over the entire analyzed period are not available in official epidemiological surveillance databases. Considering that incorrect specification of the offset in Poisson models may introduce substantive bias in rate estimates, we opted not to operationalize this strategy empirically, in order to avoid introducing structural uncertainty in the denominator that could compromise the inferential robustness of the results.

Thus, calendar year was used as a proxy variable capable of capturing the aggregated and temporally structured effect of vaccination expansion, accumulation of population immunity, changes in diagnostic practices, public health interventions, and viral evolution. This choice is coherent with the ecological study design and with the analytical objective of estimating relative risk variations at the population level over time, without the intention of mechanistic decomposition of individual risk.

All statistical analyses were performed in the R statistical environment (updated version), adopting a 5% significance level.

#### Use of Monthly and Annual Time Series

**Figure 1:**
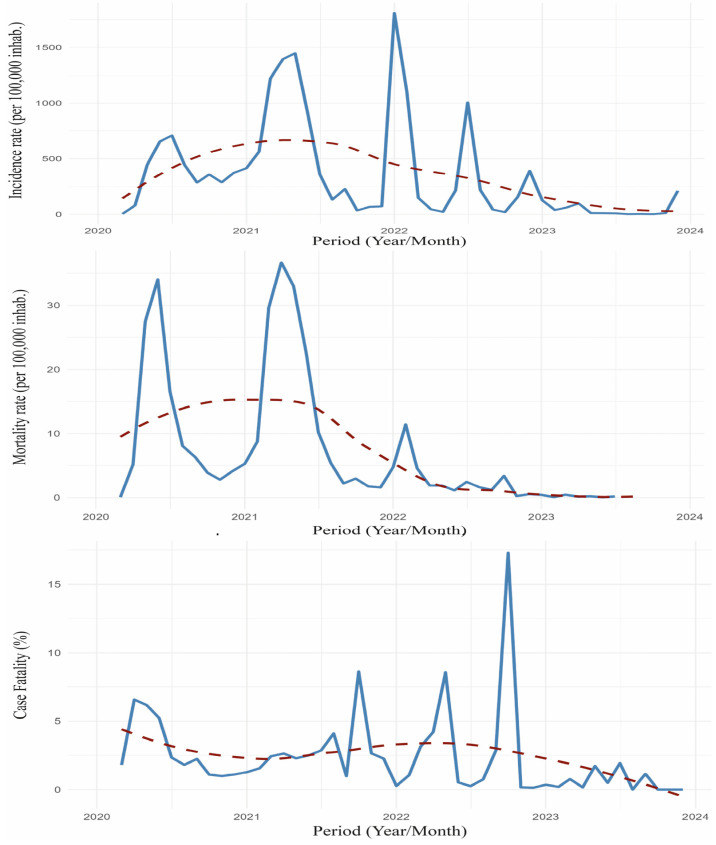
Monthly time series of Incidence Rate, Mortality Rate, and Case Fatality Ratio.

**Figure 2:**
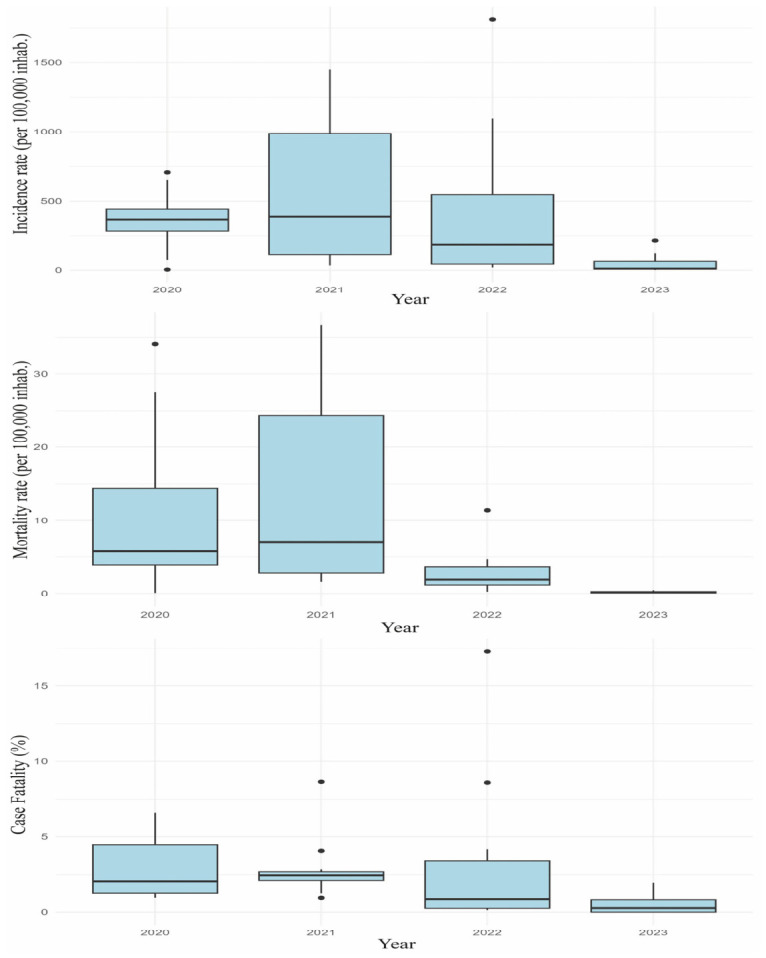
Annual distribution of monthly COVID-19 epidemiological indicators in Ceará, Brazil (2020–2023). Boxplots represent the median (horizontal line), interquartile range (box), and minimum/maximum values within 1.5 × IQR (whiskers). Black dots indicate outlier observations beyond 1.5 × IQR.

Regarding the utilization of the temporal data, (i) the monthly aggregation was used to construct the temporal epidemiological curves presented in Figure 1; and (ii) the annual aggregation, derived from the monthly distribution of the epidemiological indicators, is represented by the boxplots in Figure 2.

However, for the main inference of the study and the GLM-Poisson modeling, we opted for the annual aggregation of the data. Thus, the models used the aggregated annual numbers of confirmed COVID-19 cases and deaths as dependent variables, while the calendar year (2020–2023) was included as a categorical independent variable, using 2020 as the reference category.

Incidence and mortality rates were calculated using the annual totals of cases and deaths divided by the respective annual population estimates provided by IBGE for the state of Ceará. Case-fatality rates were calculated annually as the proportion of deaths to confirmed cases in each year. In this manner, the GLM-Poisson models estimated Rate Ratios (RRs) comparing the different aggregated epidemiological years against the baseline period of 2020.

### 2.6. Ethical Considerations

The study used exclusively secondary, anonymized, and publicly available data from official and consolidated information systems. Because it involves the use of unrestricted and aggregated data, the work is exempt from review by a Research Ethics Committee (REC), as provided for in Resolution No. 466/2012 and Resolution No. 510/2016 of the National Health Council (CNS).

Additionally, this approach complies with the most recent CNS guidelines on the use of databases for research, established in Resolution No. 738/2024. The secrecy and confidentiality of information were fully preserved, respecting ethical principles and the informational self-determination of individuals, guaranteeing the ethical rigor of scientific research.

## 3. Results

### 3.1. Descriptive Dynamics and Temporal Resolution (Monthly vs. Annual)

The evolution of the COVID-19 pandemic in Ceará, from 2020 to 2023, exhibited distinct patterns when observed at different temporal resolutions. Monthly data (Figure 1) reveal specific epidemic waves, notably the high-incidence surge in early 2022 associated with the Omicron variant. However, for the purpose of quantifying long-term risk transitions, annual aggregation (Table 1) was employed to characterize the macro-epidemiological stabilization.

While the absolute monthly peak of incidence occurred in 2022 (Figure 1), the annual average shows that 2021 remained the year with the highest sustained incidence (570.15/100,000) and mortality burden (13.34/100,000) (Table 1). The transition in 2023 is marked by a sharp decline across all indicators, representing the final stabilization phase (Figure 1, Table 1). Regarding data quality, missingness was minimal (<2% of records), and sensitivity analysis confirmed that the standardized validation procedures did not significantly alter the annual Rate Ratios.

### 3.2. Comparative Analysis of Epidemiological Indicators

To assess differences between years, we utilized the monthly distributions within each calendar year (*n* = 12 months per year, total *N* = 48) to provide the statistical power necessary for comparison (Figure 2). The annual distributions illustrated by boxplots (Figure 2) show the dispersion and outliers, particularly in 2021 and 2022.

### 3.3. Modeling the Time Trend and Relative Risk (GLM—With Poisson Distribution)

The time trend modeling, fitted by Generalized Linear Models (GLMs) with a Poisson distribution, used 2020 as the reference year and quantified the variation in risk through Rate Ratios (RRs) (Table 2).

The risk of incidence increased in 2021 (RR = 1.88; *p* < 0.01) and 2022 (RR = 1.42; *p* < 0.01) compared to 2020. However, in 2023, there was a significant reduction in the risk of new cases (RR = 0.16; *p* < 0.01), representing a decrease of approximately 84% compared to the baseline year (2020).

The assessment of mortality risk from COVID-19, comparing it to 2020 (the baseline year), showed that in 2021 the risk increased (RR = 1.47; *p* < 0.01). However, a significant drop in risk was observed in 2022, where the Relative Risk (RR = 0.32; *p* < 0.01) represented a reduction of approximately 68% in the risk of death. This downward trend became drastic in 2023, with the RR reaching 0.01 (95% CI 0.01–0.01; *p* < 0.001), indicating a marked reduction of approximately 99% in the risk of mortality from COVID-19 compared to the beginning of the pandemic.

The case fatality rate showed a reduction in 2021 (22%) and 2022 (78%). The most significant reduction occurred in 2023 (RR = 0.09; *p* < 0.01), indicating a drop of approximately 91% in the proportion of deaths among confirmed cases. The risk of death was the first component to show a significant decline, reaching an approximate reduction of 68% as early as 2022 (95% CI 0.31–0.33). This trend culminated in 2023, with an even sharper drop (95% CI 0.01–0.01), corresponding to a total reduction of 99% in the risk of mortality compared to 2020.

## 4. Discussion

This study confirms an epidemiological transition of the COVID-19 pandemic in the state of Ceará between 2020 and 2023. Ultimately, our findings demonstrate that, despite high viral circulation, the risk of severe outcomes was significantly reduced due to pharmaceutical and non-pharmaceutical interventions [25,26,27]. This transition is characterized by a profound epidemiological decoupling, where a clear dissociation emerged between sustained viral incidence and falling mortality rates from 2022 onward.

This shifting dynamic is particularly noteworthy when contrasted with the state’s baseline vulnerabilities. In line with previous ecological studies showing that the COVID-19 profile initially concentrated on elderly and vulnerable populations [17,28,29,30,31], Ceará’s trajectory was structurally tied to a low Human Development Index (HDI 0.658) and low average per capita household income [2]. This socioeconomic background created a syndemic context, where historical social inequalities and viral circulation amplified health system strain, thereby limiting the immediate effectiveness of legal control measures such as Brazilian Law No. 13,979/2020 [12,32].

Remarkably, despite these persistent structural barriers and early evidence of systemic fragility and social insecurity [5], the state successfully transitioned to a low-risk scenario by 2023. This shift underscores a critical mechanism driven by the capillarization of the Brazilian Unified Health System (SUS), where a progressive public health response, anchored by primary healthcare professionals, managed to restrict demand, maintain service functions, and effectively mitigate the impact of social determinants over time [14].

Nevertheless, this mitigation process also exposed sharp regional challenges [14]. The prolonged stability of incidence until 2022 reflects the spread of the Omicron variant within a highly mobile yet unequal population [33,34,35,36], differing substantially from European [37] and Asian [38] settings. These unique dynamics underscore the role of vaccination in Ceará as a crucial harm reduction strategy, which protected a highly vulnerable population against severe outcomes without necessarily halting short-term transmission [39].

To capture these complex shifts with statistical rigor, we utilized Generalized Linear Models (GLMs) with a negative binomial or quasi-Poisson specification to address overdispersion in the count data, thereby ensuring greater accuracy and reducing bias compared to a standard Poisson distribution. Furthermore, the estimation of Rate Ratios (RRs) offers a clear, actionable metric for healthcare managers and policymakers.

Despite this robust analytical framework, certain methodological limitations inherent to ecological design require caution. First, the ecological nature of the data introduces the risk of ecological fallacy, preventing causal inferences at the individual level; consequently, temporal associations and relationships with interventions were used contextually to understand macro-patterns and rely on existing literature rather than individual confounding controls.

Secondly, the reliance on secondary data from the National Disease Notification System (SINAN) and the Mortality Information System (SIM) introduces notification biases. Chronic underreporting, exacerbated after 2022 by reduced testing and altered case definitions, underestimated the Incidence Rate, potentially inflating the 84% drop observed in 2023, while paradoxically overestimating the Case Fatality Rate. Nevertheless, the precipitous 99% drop in mortality risk in 2023 is of such magnitude that it likely represents a true biological stabilization rather than a mere surveillance artifact.

Because actual prevalence was undoubtedly higher than reported, ongoing epidemiological and genomic surveillance remains vital to monitor how COVID-19 operates within structurally vulnerable territories. Ultimately, these findings demonstrate that ecological studies, when modeled accurately, serve as critical sentinel tools to detect shifts in disease patterns, enabling agile and timely responses to future resurgences.

## 5. Conclusions

Surveillance indicators in Ceará show a clear transition toward reduced COVID-19 severity between 2020 and 2023, with mortality declining earliest and most sharply. These patterns are consistent with combined effects of vaccination, accumulated immunity, and strengthened clinical and primary-care capacity, although the ecological design and absence of age-stratified or variant-specific data limit causal attribution. Compared with existing regional analyses, this study contributes a longer-term, population-level view of epidemiological stabilization in a structurally vulnerable setting. Taken together, the findings underscore the importance of equitable vaccine distribution, resilient primary healthcare, and sustained surveillance systems, while highlighting the need for future work integrating immunization and sociodemographic data to clarify the mechanisms driving these improvements.

## Figures and Tables

**Table 1:** Epidemiological indicators of COVID-19 in Ceará (2020–2023) based on annual aggregation.

Indicator	2020 (Annual Average)	2021 (Annual Average)	2022 (Annual Average)	2023 (Annual Average)
Incidence Rate (per 100,000 inhabitants)	363.49	570.15	430.57	48.47
Mortality Rate (per 100,000 inhabitants)	10.48	13.34	2.92	0.20
Case Fatality (%)	2.93	2.84	3.26	0.56

**Table 2:** Risk modeling and Rate Ratios (RRs) for COVID-19 indicators in Ceará per Year of study, using 2020 as the reference year for COVID-19 in the state of Ceará.

Indicator	Year	Rate Ratio (RR)	IC 95% Inferior	IC 95% Superior	*p*-Value	Variation in Risk
Incidence (per 100,000 inhabitants)	2020 (ref.)	1	-	-	-	-
	2021	1.88	1.87	1.88	<0.01	Increase (88%)
	2022	1.42	1.41	1.42	<0.01	Increase (42%)
	2023	0.16	0.15	0.16	<0.01	Reduction (84%)
Mortality (per 100,000 inhabitants)	2020 (ref.)	1	-	-	-	-
	2021	1.47	1.43	1.51	<0.01	Increase (47%)
	2022	0.32	0.31	0.33	<0.01	Reduction (68%)
	2023	0.01	0.01	0.01	<0.01	Reduction (99%)
Case Fatality (%)	2020 (ref.)	1	-	-	-	-
	2021	0.78	0.76	0.80	<0.01	Reduction (22%)
	2022	0.22	0.21	0.23	<0.01	Reduction (78%)
	2023	0.09	0.07	0.10	<0.01	Reduction (91%)

## Data Availability

Data were extracted from: https://covid.saude.gov.br/ (accessed on 10 January 2024).

## Abbreviations

SARS-CoV-2	Severe Acute Respiratory Syndrome Coronavirus 2
REC	Ethics Committee
NHC	National Health Council
GLM	Generalized Linear Model
RR	Rate Ratio
